# When do drugs trigger criminal behavior? a machine learning analysis of offenders and non-offenders with schizophrenia and comorbid substance use disorder

**DOI:** 10.3389/fpsyt.2024.1356843

**Published:** 2024-03-07

**Authors:** Ewa-Maria Bender, Lena Machetanz, Roland von Känel, Sebastian Euler, Johannes Kirchebner, Moritz Philipp Günther

**Affiliations:** ^1^ Department of Consultation-Liaison Psychiatry and Psychosomatic Medicine, University Hospital Zurich, University of Zürich, Zurich, Switzerland; ^2^ Department of Forensic Psychiatry, University Hospital of Psychiatry Zurich, Zurich, Switzerland; ^3^ Privatklinik Meiringen, Willigen, Meiringen, Switzerland

**Keywords:** forensic psychiatric patients, non-forensic patients, offending, schizophrenia spectrum disorder, substance use disorder, supervised machine learning, explorative analysis

## Abstract

**Introduction:**

Comorbid substance use disorder (SUD) is linked to a higher risk of violence in patients with schizophrenia spectrum disorder (SSD). The objective of this study is to explore the most distinguishing factors between offending and non-offending patients diagnosed with SSD and comorbid SUD using supervised machine learning.

**Methods:**

A total of 269 offender patients and 184 non-offender patients, all diagnosed with SSD and SUD, were assessed using supervised machine learning algorithms.

**Results:**

Failures during opening, referring to rule violations during a permitted temporary leave from an inpatient ward or during the opening of an otherwise closed ward, was found to be the most influential distinguishing factor, closely followed by non-compliance with medication (in the psychiatric history). Following in succession were social isolation in the past, no antipsychotics prescribed (in the psychiatric history), and no outpatient psychiatric treatments before the current hospitalization.

**Discussion:**

This research identifies critical factors distinguishing offending patients from non-offending patients with SSD and SUD. Among various risk factors considered in prior research, this study emphasizes treatment-related differences between the groups, indicating the potential for improvement regarding access and maintenance of treatment in this particular population. Further research is warranted to explore the relationship between social isolation and delinquency in this patient population.

## Introduction

Violent and aggressive behaviour is commonly perceived to be more frequent in patients with Schizophrenia Spectrum Disorder (SSD). This perception is supported by research, such as that conducted by Fazel et al (1). In their systematic review, they find odds ratios (OR) for violence of individuals with schizophrenia between 1 and 7 in men and between 4 and up to 29 in women compared to their general populations. However, the extent to which either the psychopathology inherent to SSD or factors beyond the diagnosis influence these figures is an ongoing debate.

Some studies show an association between violence and certain psychopathological factors of SSD such as positive symptoms and lack of insight (2, 3). However, while some literature suggest an association between threat-control override symptoms and increased violent tendencies (4–6), Witt et al.’s systematic review and meta-analysis has found no significant correlation (3). Rather, they emphasize many factors beyond the diagnosis of SSD to be strongly associated with violence. These include static factors, such as sex, age, socio-economic status, childhood maltreatment, conduct disorder, recent drug misuse (OR 2.2), recent substance misuse (OR 2.9), recent alcohol misuse (OR 2.2), and criminal history factors (OR 3.1) (3, 7), but also more dynamic factors, which seem potentially modifiable through treatment. Examples of these factors are hostile behaviour (OR 1.5), non-adherence with psychotherapy (OR 6.7), non-adherence with medication (OR 2.0) and education (3).

The important role of comorbid Substance Use Disorder (SUD) concerning violent behaviour is also highlighted in Fazel et al.’s systematic review (1). They find an OR for violence of 8.9 for patients diagnosed with SSD and SUD, whereas patients with SSD but without SUD have a significantly lower OR of 2.1. Interestingly, patients with SSD and comorbid SUD did not differ greatly from substance-using individuals without a diagnosis of SSD. The correlation between SUD and violence is also supported by the systematic review of Zhong et al. (8). They find that individuals with SUD have a 4-10 times higher likelihood of violence compared to the general population. They also find an increased risk of violence for all studied substances individually. However, because few studies investigated the different substances independently, it was not possible to draw definitive conclusions about the hierarchy of these substances in terms of their association with violence.

There may also be factors, e.g. genetic or developmental, that affect SUD or violence or both simultaneously (9). When comparing patients with SSD and comorbid SUD with their SSD-unaffected siblings, the difference in risk was less pronounced. SSD-unaffected siblings also showed higher rates of SUD than the general population. This suggests that there are confounders that may influence SSD, SUD and violence simultaneously.

Although SSD is a low prevalence disorder, patients with this diagnosis make up a large proportion of patients in forensic psychiatry (10, 11). 41.7% of patients with SSD suffer from comorbid SUD (12). Given the frequent co-occurrence of SSD and SUD, as well as the statistical impact of SUD on the risk of violence, it is important to examine this subgroup specifically when considering possible factors influencing delinquency and violence. Better understanding of the underlying causes of violent and offending behaviour is imperative for devising effective preventative and therapeutical interventions. Research comparing individuals with SSD who are offenders to those who are not is limited, and is mostly conducted in populations with mixed diagnoses and small case numbers (13, 14). In this study we compare patients with SSD and comorbid SUD in a forensic setting (offender patients) and a general psychiatric setting (non-offender patients). The non-offender patients in this study were in an inpatient psychiatric treatment, mostly with an already established pharmacotherapy upon admission and in majority with prolonged or chronic courses of the disorder, which makes them a suitable comparison group to the offender group, also in inpatient psychiatric treatment. The objective of this study is to explore distinguishing factors between offenders and non-offenders diagnosed with SSD and comorbid SUD using supervised machine learning.

## Materials and methods

### Population

The first half of the study group consisted of 269 offender patients (oP) with a diagnosis of SSD (F20.0-F25.9 acc. to ICD-10; 295.0-295.9 acc. to ICD-9) and a co-morbidity of any kind of substance use disorder regardless of severity or type of substance (F10.0-F19.9 acc. to ICD-10) who had all court-mandated inpatient treatment at the Centre for Inpatient Forensic Therapies of the University Hospital of Psychiatry Zurich, Switzerland (15, 16). In Switzerland, court-ordered treatment is possible if an expert report establishes a close connection between the perpetration of a criminal offence and a mental illness, i.e. the offence is an expression of the underlying symptomatology (17). Offenses that can lead to such an institutionalization in our sample were either violent or non-violent in nature, or, in case of several offenses, both.

The second half of the study group held 184 non-offender patients (noP) with the same diagnostic inclusion criteria, who had been in inpatient treatment at the Centre for Integrative Psychiatry of the University Hospital of Psychiatry Zurich. Patients are administered either on a voluntary basis or as compulsory admissions due to imminent danger of self-harm or harm to others (so-called “Fürsorgerische Unterbringung/FU”) (18). We considered this sample suitable for comparison due to the following reasons: First, it predominantly also consisted of individuals with chronic and prolonged SSD. Second, upon admission, it already had an established pharmacotherapy, often being transferred from acute psychiatric wards—a pattern shared with the majority of forensic patients, who were initially treated in a prison or custodial setting.

Both groups included approximately the same percentage of male and female patients.

### Source of data

Ethics approval for the study was provided by the cantonal ethics board of Zurich, Switzerland (BASEC No. 2014-0480 and PB_2016-01903). Data stemmed from the case files of the patients described above. These files included professionally documented medical histories, psychiatric/psychologic inpatient and outpatient reports, extensive progress reports by health care staff of several disciplines, including doctors, nurses and special therapists such as occupational or art therapists. In the oP population, the files naturally also included testimonies, court proceedings and data regarding previous imprisonments and detentions.

### Data extraction

Data extraction followed the principles of directed qualitative content analysis (19). The case files were screened for the desired information by an experienced psychiatrist, using a standardized rating protocol developed by an expert panel with other researchers and clinicians with both forensic and general psychiatric expertise. Independently, another researcher encoded a random subsample of 10% of all cases to assess inter-rater reliability.

### Outcome variable

The outcome variable was dichotomized, with “noP: TRUE” for non-offender patients vs. “noP: FALSE” for offender patients. In further analysis, the former was defined as the positive class.

### Selection of predictor variables

Predictor variables included in the analysis were selected based on previous findings. A list of all 175 predictor variables and their description can be found in the **Appendix**. The detailed coding protocol, with definitions of all variables used in this project was published in august 2022 (20).

### Data analysis using supervised machine learning

**Figure 1:**
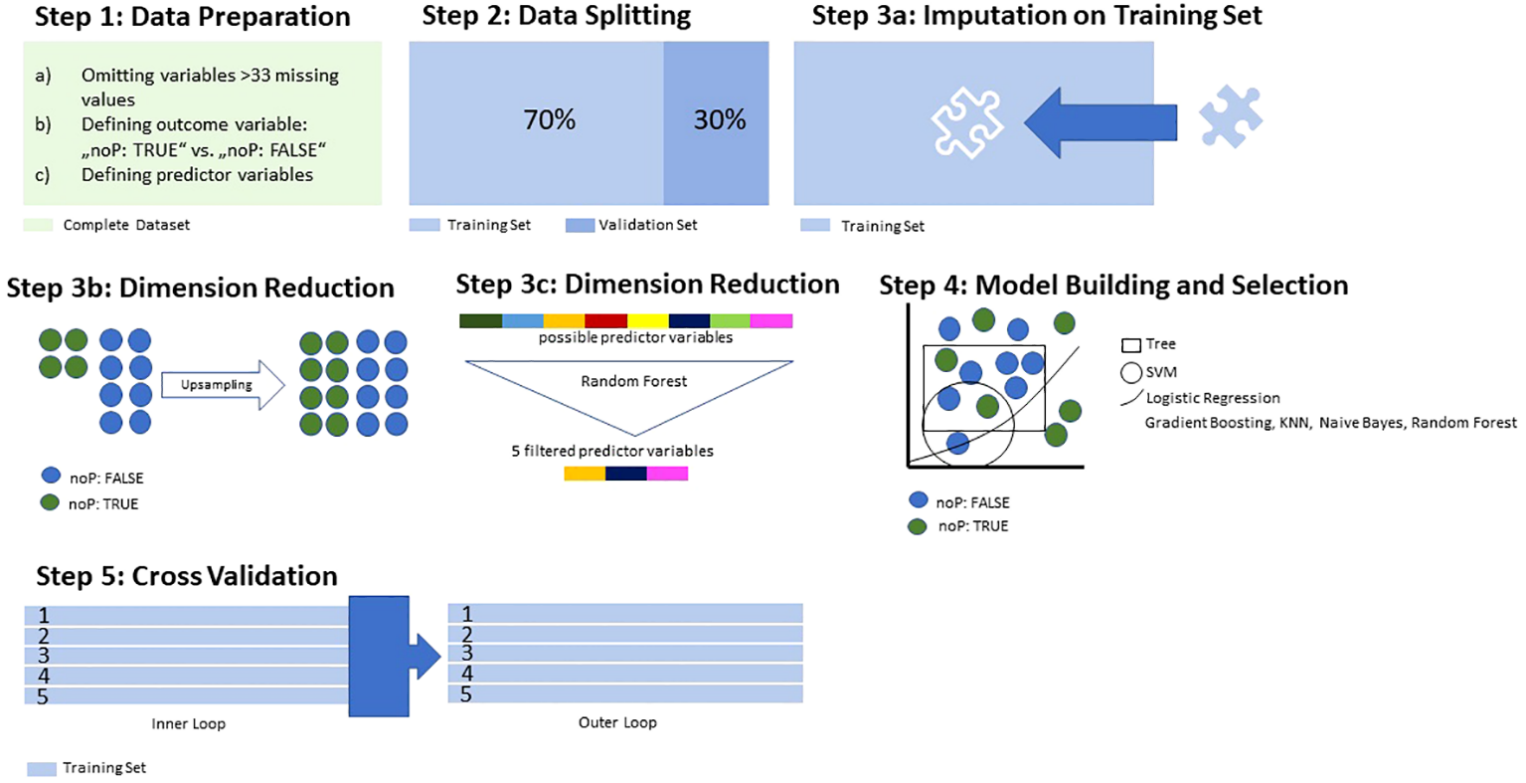
Data Processing and Training.

All the steps were performed using R version 3.6.3. (R Project, Vienna, Austria) and the MLR package v2.171 (Bischl, Munich, Germany). Balanced accuracy was calculated using MATLAB R2019a (MATLAB and Statistics Toolbox Release 2012, The MathWorks, Inc., Natick, MA, USA) with the add-on “computing the posterior balanced accuracy” v1.0. First, all raw data were processed for ML (see **Figure 1**, Step 1): All categorical variables were converted to binary code, while continuous and ordinal variables were not adjusted. Variables with >33% of missing values were excluded from further analyses. As a next step, we divided the complete database consisting of both noP and oP into a training subset and a validation subset, with the latter remaining untouched for now, and the former being used for dimensionality reduction and model building and selection (**Figure 1**, step 2). The training subset contained 70%, the validation subset 30% of all cases. To enable the flexible application of several ML algorithms, imputation of missing values was carried out and imputation weights saved for later were reused on the validation subset (**Figure 1**, step 3a). As the outcome variable “noP: TRUE” vs. “noP: FALSE” was unevenly distributed, we carried out a random up-sampling to create a more balanced outcome (**Figure 1**, step 3b). To counteract overfitting and maintain acceptable computing times in the model building process, a reduction in dimensionality was performed applying a random-ForestSCR algorithm (**Figure 1**, step 3c). This step was performed up to the point where the AUC did not improve by >5% when adding another dimension, which left a set of 5 variables. This concluded the preprocessing.

For discriminative model building, the following algorithms were applied to the training set: logistic regression, trees, random forest, gradient boosting, KNN (k-nearest neighbour), support vector machines (SVM), and naive Bayes (**Figure 1**, step 4). The performance of each model was then calculated and evaluated regarding the balanced accuracy, the goodness of fit (with the receiver operating characteristic as measurement) as well as its sensitivity, specificity, positive and negative predictive value. The model with the highest area under the curve (AUC) was selected for the final model validation on the validation subset. Lastly, we applied a so-called nested-resampling approach for the prevention of overfitting, with an inner loop performing the steps 3a-4 within 5-fold cross-validation, and an outer loop with performance evaluation also embedded in 5-fold cross-validation (**Figure 1**, step 5). This was another measure for the prevention of overfitting.

**Figure 2:**
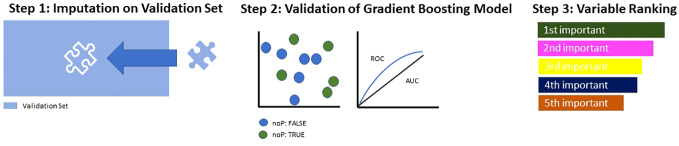
Model Building and Testing on Validation Set.

Then, the selected model was tested on the validation subset previously stored aside: First of all, the imputation weight saved earlier were applied for imputation of missing values within the validation subset (**Figure 2**, step 1). Then, the most suitable model was applied for validation, and its performance measures were quantified (**Figure 2**, step 2).

Finally, the identified predictor variables were tested for multicollinearity to avoid interdependence between them, and then ranked in accordance with their relative influence within the model (**Figure 2**, step 3).

## Results

### Descriptive data

The primary project sample consisted of 370 offending patients (oP) with a diagnosis of SSD (F2x acc. to ICD-10, respectively, 295.x acc. to ICD-9) and 370 non-offending patients (noP) with the same diagnosis of SSD. In this study, only those with comorbid substance use disorder were considered, resulting in 269 individuals in the forensic group and 184 in the non-forensic group. The difference in mean age at admission between the two groups is relatively small (oP = 32.2 years, SD= 9.1; noP = 32.4 years, SD = 10.4). Both groups consist of predominantly single males. All patients were diagnosed with SSD and a co-diagnosis of SUD (F1x acc. to ICD-10, chapters 303-305 acc. to ICD-9). The mean age at which the F2x diagnosis was given is slightly higher for oP (26.2 years) compared to noP (24.0 years). While cannabis use disorder is relatively more common in noP (90.2% vs. 82.9% in oP), Other substance use disorders are more common in oP. oP have a higher percentage of opioid use disorder (38.2 vs. 21.8%), cocaine use disorder (45.4% vs. 28.7%) and stimulant use disorder (30.5% vs. 18.4%). A history of alcohol or drug abuse during the patients childhood or youth is more prevalent among noP (61.1%) than oP (52.4%). A notable discrepancy is observed in the country of birth, where 48.7% of oP are born in Switzerland compared to 71% of noP. Regarding education, 25.7% of oP have no graduation from compulsory school, contrasting with 5.5% in the noP group. Regarding unemployment, 45.9% of oP were non-workers for most of the time they were of working age, whereas 57.3% of noP fall into this category (see **Table 1**).

**Table 1:** Basic data.

Variable description	Forensic patients (oP)		Non forensic patients (noP)	
	n/N (%)	mean (SD)	n/N (%)	mean (SD)
Age at admission		32.2 (9.1)		32.4 (10.4)
Gender male	253/269 (94.1)		177/183 (96.7)	
Country of birth Switzerland	131/269 (48.7)		130/183 (71)	
Single	228/265 (86)		158/184 (85.9)	
No graduation from compulsory school	63/245 (25.7)		9/164 (5.5)	
Nonworker for most of the time	105/229 (45.9)		94/164 (57.3)	
Diagnosis: Schizophrenia	214/269 (79.6)		161/184 (87.5)	
Age at F2x diagnosis		26.2 (7.9)		24.0 (7.0)
Cannabis use disorder	223/269 (82.9)		157/174 (90.2)	
Opioid use disorder	103/269 (38.3)		38/174 (21.8)	
Cocaine use disorder	122/269 (45.4)		50/174 (28.7)	
Stimulants use disorder	82/269 (30.5)		32/174 (18.4)	
alcohol or drug abuse in patient’s childhood/youth?	108/206 (52.4)		69/113 (61.1)	

n, subgroup with characteristics; N, total study population; SD, Standard deviation.

### Model calculation and performance measures

By comparing the performance of seven different algorithms on the training set, Gradient Boosting was identified as the most reliable algorithm to identify oP. Gradient Boosting showed a balanced accuracy of 77.5% and an AUC of 0.88. with a specificity of 91%, meaning that oP were correctly identified in 9 out of 10 cases in the training set (See **Table 2**).

**Table 2:** Machine learning models and performance in nested cross-validation.

Statistical Procedure	Balanced Accuracy (%)	AUC	Sensitivity (%)	Specificity (%)	PPV (%)	NPV (%)
Logistic Regression	75.30	0.86	63.8	86.7	76.	77.5
Tree	76.00	0.86	66.70	85.3	78.3	78.8
Random Forest	76.8	0.86	63.1	94.3	88.1	79.1
**Gradient** **Boosting**	**77.5**	**0.88**	**64**	**91**	**82.6**	**78.9**
KNN	76.4	0.81	92.3	42.5	52.8	90.2
SVM	74.6	0.86	63.2	86.2	77.1	77.7
Naive Bayes	77	0.86	62.8	91.2	82.8	78.3

AUC, area under the curve (level of discrimination); PPV, positive predictive value; NPV, negative predictive value; KNN, k-nearest neighbors; SVM, support vector machines.

Bold indicates the best performing model and its values.

In the final analysis 175 variables were included. 5 predictor variables emerged as most indicative. These variables were: Failures during opening, medication compliance (in psychiatric history), social isolation in past, antipsychotics prescribed (in psychiatric history) and outpatient psychiatric treatment(s) before current hospitalisation (See **Table 3**). “Failures during opening” refers to rule violations during a permitted temporary leave from an inpatient ward or during the opening of an otherwise closed ward, including staying away from the ward longer than allowed, not returning at all, the prohibited consumption of substances or any other violation of previously agreed upon conditions. The definition of all five variables can be found in the **Appendix**.

**Table 3:** Absolute and relative distribution of relevant predictor variables.

Variable code	Variable description	Forensic patients (oP) n/N(%)	Non-Forensic patients (noP) n/N(%)
PH18a	Outpatient psychiatric treatment(s) before current hospitalisation	127/241 (52.7)	**142/162 (87.7)**
PH23a	Antipsychotics prescribed (in psychiatric history)	162/269 (60.2)	**168/181 (92.8)**
PH23u	Medication compliance (in psychiatric history)	15/145 (10.3)	**72/149 (48.3)**
DZ12	Failures during opening	53/173 (30.6)	**99/167 (59.3)**
S9i	Social Isolation in past	**124/263 (47.1)**	32/162 (19.8)

Higher expression of the specific variable in a group is indicated by bold font.

### Final model performance

Upon application to the validation subset the gradient boosting algorithm demonstrated a balanced accuracy of 76.6% and an AUC of 0.84. The specificity achieved was 87.7%, nearly mirroring the performance on the training subset (see **Table 4**). Notably, due to variations in data processing between the validation and training set, the algorithm’s performance metrics were slightly reduced, but still able to correctly identify over 4 out of 5 cases.

**Table 4:** Final Gradient Boosting model performance measures on validation subset.

Performance measures	% (95% CI)
**Balanced** **Accuracy**	76.6 (71 -84.7)
**AUC**	0.84 (0.77-0.91)
**Sensitivity**	65.5 (51.3 – 77.4)
**Specificity**	87.7 (78 – 93.6)
**PPV**	83.6 (75.1 – 89.8)
**NPV**	75.9 (66.7 – 83.3)

AUC, area under the curve (level of discrimination); PPV, positive predictive value; NPV, negative predictive value.

### Ranking of the predictor variables by their indicative power

**Figure 3:**
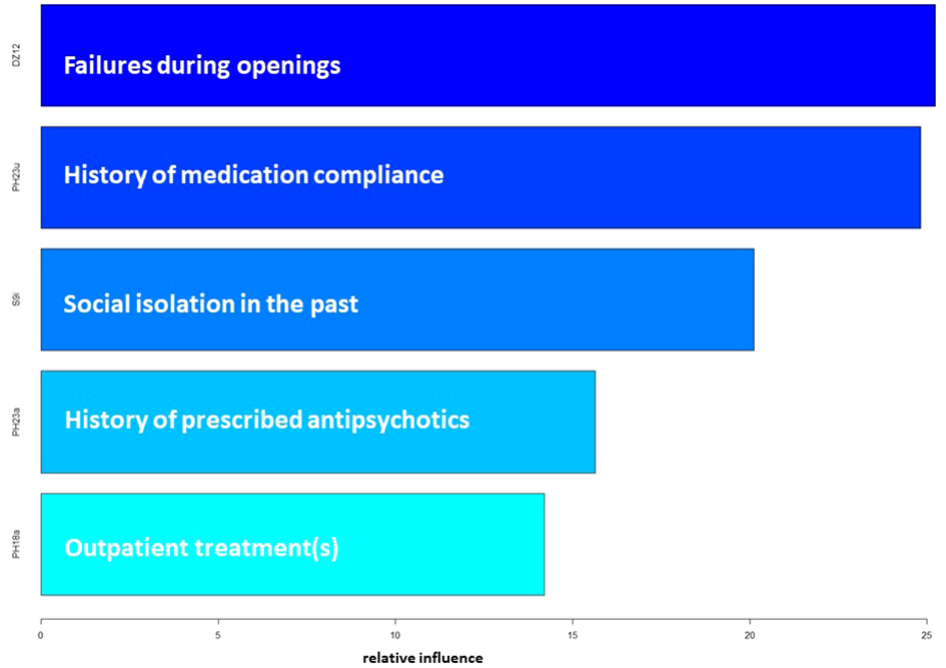
Ranking of the Predictor Variables in the gradient boosting model.

When ranked by their indicative power, failures during opening was found to be the most influential distinguishing factor, closely followed by medication compliance (in psychiatric history). Following in succession were social isolation in the past, antipsychotics prescribed (in psychiatric history), and outpatient psychiatric treatments before the current hospitalization (See **Figure 3**).

## Discussion

Prior literature emphasises the significance of comorbid SUD as one of the most relevant factors contributing to the increased risk of violence in patients with SSD (9). This paper aims to gain a better insight into this already described high-risk group. This is done by exploring how patients who have come into contact with the law differ from those who have not. Due to the substantial data and group size it was possible to use supervised machine learning with its benefit of being able to sift through larger datasets and uncover hidden patterns and relationships that may not be readily apparent through traditional statistical approaches. Our model was able to determine 5 variables that are best suited to distinguish between the two groups of oP vs. noP. Forensic patients stood out in terms of their treatment history and in the prevalence of experiencing social isolation. We found that oP are less likely to have been prescribed antipsychotics in the past, have a significantly poorer history of compliance, have been less connected to outpatient services and were more likely to experience social isolation.

Failures during opening, more prevalent in noP, emerged as the variable with the highest indicative power. Failures during opening refers to rule violations during a permitted temporary leave from an inpatient ward or during the opening of an otherwise closed ward. While this result may be surprising at first, considering that oP have a history of breaking the law and therefore breaking rules seems more likely, this result is mostly due to two factors: First, failures during opening were likely to be less observed in oP due to their lower exposure to inpatient treatment in the past, limiting the opportunity for such observations. Second, there are structural differences between the current treatments between the groups. Forensic clinics have higher security measures, fewer opportunities for rule violations, and rule violations can have significant consequences for the patient, e.g. legal consequences (21). This means that there is both less opportunity to engage in such behaviour as well as a greater disincentive.

Compliance with medication regimen (in patient history) was the second strongest indicator to distinguish the groups. Non-compliance was significantly higher in oP. This is consistent with literature associating medication non-compliance with higher rates of violent and non-violent crimes (3, 22, 23). Medication non-compliance is a common phenomenon in patients with SSD and is even more common in patients with comorbid SUD (23, 24). Compliance is influenced by various factors. They include the medication itself, its dosage and potential side effects, as well as family and social support and patients’ attitudes towards therapy – all of which can impact a patient’s conscious and subconscious willingness to adhere to the prescribed regimen (24, 25).

Closely related to non-compliance with medication, results indicate oP had more frequently no antipsychotics at all prescribed in the past. This is concerning given the pivotal role of antipsychotics in managing symptom severity in SSD and their demonstrated impact on reducing aggression (26).

This finding can be explained partially by lack of prior contact with the health care system, partially by more resistance against even the prescription of antipsychotics, both of which may be related to lack of insight into the disease or lack of attention from society as a whole.

This leads to social isolation as a noteworthy factor more predominant in the oP-group. Studies on risk factors for violent behaviour in patients with SSD have largely ignored social isolation. Instead, related factors, such as social withdrawal behaviour or living alone (2, 3) are explored, but provide insufficient information on factual social isolation. In addition to the open question of how social isolation in patients with SSD might be related to delinquency, the interplay between social isolation and SSD itself has not yet been fully explained. In particular, whether isolation precedes SSD, possibly also as a triggering factor, or whether social isolation is rather a consequence of the diagnosis, remains to be determined (27). While our results do not allow for causal inferences, they do suggest social isolation may not have been sufficiently considered as a risk factor in previous studies on violence. Social interaction, be it a professional interaction with a therapist or more casual, may be an anchor against drifting into psychosis, substance use or violence.

Zhong et al. argue in their systematic review that, while future research is needed, there is reason to hypothesize that different substances pose varying risks for violent behavior (8). Although our descriptive data indicates oP and noP favour different illegal substances on average, these differences were not sufficient for the model to distinguish between the groups. However, important information about substance use was also missing, as we only had dichotomous values on whether there was use/dependence or not and many patients used several substances.

The literature describes an association between various domains of risk factors and violence, including psychopathology, substance use factors, childhood experiences, treatment-related, premorbid and sociodemographic factors (3, 28–30). Considering the diverse nature of influencing factors, it is notable that this study reveals an emphasis on treatment-related factors and introduces social isolation as a variable of interest, which has not received sufficient attention to date.

These findings emphasise the importance of low-threshold access to psychiatric and psychological help and that there is a subgroup of high-risk patients who face difficulties in obtaining and maintaining this help.

### Strengths and limitations

A frequent limitation of retrospective data analysis also inherent to this study is the lack of temporal linkage between predictor variables and outcome variable. In addition, certain variables of interest, e.g. childhood variables, had to be excluded from the statistical analysis due to the substantial number of missing values in the noP-group. While our group sizes are large, considering the specific setting and comorbidity of SUD, the dataset is rather small for a machine learning approach, thereby possibly containing risks of overfitting or biased performance measurements (31). While appropriate methods were used to counteract these issues, further validation of the model is needed. Moreover, the use of dichotomous variables for complex information, such as substance use, may lead to an incomplete understanding of the subject. On the other hand, some dichotomization of data is needed to allow for quantitative advanced statistical approaches.

On that note, the advanced statistical approach we chose reached a level of complexity difficult to encapsulate in detail and clarity within the boundaries of a non-statistical publication. However, readers interested in further detail are encouraged to contact the authors, as the brevity of the methods section should not hamper reproducibility of results (32).

Conceptually more complex variables, such as disease insight, may be more prone to misjudgement by psychiatrists exploring patients and be less suitable for statistical analysis. This could also explain why this variable did not emerge as a distinguishing factor between the two groups.

It is important to note that our population is predominantly male, a predominant problem in research on SSD. However, this gender difference is a depiction of the real situation in treating SSD in forensic psychiatry in Switzerland.

Since we wanted to study the forensic psychiatric sample in its entirety as far as possible, we also did not exclude offender patients from the sample based on type or severity of their crime, although prior research has demonstrated a correlation between SUD and criminal behavior (33).

## Conclusion

This study aimed to provide deeper insights into the high-risk population of patients with SSD and comorbid SUD and was able to identify key variables distinguishing between offender and non-offender patients. Comorbid SUD is one of the most important factors that is positively related to criminal behaviour according to previous literature. It is therefore particularly interesting and important to study the differences between offender and non-offender patients with this comorbidity. The exploratory nature of this work does not allow conclusions to be drawn about causal relationships, but it does speak to the likely importance of different variables and suggests areas for future research.

Among the various variables considered as risk factors in prior literature, we find that the most indicative factors are related to treatment issues in the patients past, such as a lack of previous outpatient treatment, non-compliance of medication and no prescription of antipsychotics in the past. Additionally, the results shift focus on social isolation, a factor having received little attention regarding offending behaviour in patients with SSD to date. This novel insight calls for further research into the relationship between social isolation and delinquency in patients with SSD. On a clinical level, our findings suggest more focus on improving access to treatment services and on reasons behind treatment non-compliance and antipsychotic prescription lethargy.

## Data availability statement

The raw data supporting the conclusions of this article will be made available by the authors, without undue reservation.

## Ethics statement

The studies involving humans were approved by The cantonal ethics board of Zurich, Switzerland (BASEC No. 2014-0480 and PB_2016-01903). The studies were conducted in accordance with the local legislation and institutional requirements. The ethics committee/institutional review board waived the requirement of written informed consent for participation from the participants or the participants’ legal guardians/next of kin because Participants difficult to reach or dead.

## Author contributions

EB: Data curation, Formal analysis, Investigation, Methodology, Project administration, Visualization, Writing – original draft, Writing – review & editing. LM: Formal analysis, Methodology, Software, Supervision, Validation, Visualization, Writing – review & editing. Rv: Resources, Supervision, Writing – review & editing. SE: Resources, Supervision, Writing – review & editing. JK: Conceptualization, Data curation, Formal analysis, Investigation, Methodology, Resources, Software, Supervision, Validation, Writing – review & editing. MG: Conceptualization, Data curation, Investigation, Methodology, Project administration, Resources, Supervision, Validation, Visualization, Writing – original draft, Writing – review & editing.
