# Supplementary material for: When do drugs trigger criminal behavior? a machine learning analysis of offenders and non-offenders with schizophrenia and comorbid substance use disorder
Source: Front Psychiatry. 2024 Mar 7;15:1356843. doi: 10.3389/fpsyt.2024.1356843 (PMC10954830; doi:10.3389/fpsyt.2024.1356843)
Supplement: Supplementary file 1 [file DataSheet_1.docx]

**Supplementary Material:
When do Drugs trigger Criminal Behavior? A Machine Learning Analysis of Offenders and Non-Offenders with Schizophrenia and Comorbid Substance Use Disorder**

Ewa-Maria Bender^1^, Dr. med. Lena Machetanz^2^, Prof. Dr. med. Roland von Känel^1^, PD Dr. med. Sebastian Euler^1^, PD Dr. med. Johannes Kirchebner^2^, PD Dr. med. Moritz Philipp Günther^1,3^

^1^Department of Consultation-Liaison Psychiatry and Psychosomatic Medicine, University Hospital Zurich, University of Zurich, Zurich, Switzerland

^2^ Department of Forensic Psychiatry, University Hospital of Psychiatry Zurich, Zurich, Switzerland

^3^ Privatklinik Meiringen, Willigen, Meiringen, Switzerland

**Description and definition of the relevant predictor variables**

| Variable code | Variable description | Definition (1) |
| --- | --- | --- |
| PH18a | Outpatient psychiatric treatment(s) before  current hospitalisation | Yes, if he/she had visited a mental health care provider (psychologist and/or psychiatrist) as an outpatient at any time before the investigated offence, regardless of the duration of said treatment |
| PH23a | Antipsychotics prescribed  (in psychiatric history) | Yes, if he/she had been prescribed any antipsychotic medication at any time before the investigated offence |
| PH23u | Medication compliance (in psychiatric history) | Yes, if he/she, mental health professionals and trusted private persons (e.g., close family members) had not reported/documented a lack of compliance/adherence to any antipsychotic medications at any time before the investigated offence AND if mental health professionals and trusted private persons (e.g., close family members) had not had reasonable grounds for suspecting that the patient lacked medication compliance/adherence to any antipsychotic medications at any time before the investigated offence |
| DZ12 | Failures during opening | ‘Failures during opening’ refers to a lack of obedience to any agreed rules while having a permission to temporarily leave an inpatient ward or during the opening of an (otherwise closed) inpatient ward, which includes any of the following …   - staying away from the ward for longer than allowed (including no return at all) - the prohibited consumption of substances (including alcohol and prescription medications) outside of the station - any other violation of agreed rules while having a permission to leave an inpatient ward or during the opening of an (otherwise closed) inpatient ward |
| S9i | Social Isolation in past | Yes, if he/she had suffered from social isolation for a period of at least 1 year before admission to the referenced forensic hospital Note: Signs of social isolation include ‘’small social networks,  infrequent social contacts, absence of confidante connections,  living alone, and lack of participation in social activities’’ (2, p.2042) |

**Description of predictor variables**

| Variable code | Variable description |
| --- | --- |
| SD1 | Age at admission? |
| SD2 | Gender? |
| SD6a | Living situation: (at time of the investigated offence) - psychiatric living measure |
| SD6b | Living situation: (at time of the investigated offence) - complementary facility |
| SD6g | Living situation: (at time of the investigated offence) - homeless |
| SD7a | Highest graduation: (at time of the investigated offence) - no compulsory school |
| SD8a | Learned profession: no job learned |
| SD9 | Is the patient a nonworker (at time of the investigated offence)? |
| SD11 | Is the patient a nonworker (most time executed)? |
| SD18a | Who was/is the legal guardian - birth parents? |
| SD18g | Who was/is the legal guardian - foster parents? |
| SD18h | Who was/is the legal guardian - child home? |
| CJ8 | Was any professional help by a psychiatrist/psychologist sought in the patients childhood/youth? |
| CJ9a | Was the patient diagnosed for ADHS/POS in his youth childhood/youth? |
| CJ17 | Loss of a parent/caregiver in the patients in his childhood/youth? |
| CJ18 | Early separation from the family/caregivers in his youth childhood/youth? |
| CJ20 | Heavy mental illness of family/caregivers in the patients childhood/youth? |
| CJ27 | Physical illness of the patient in his youth childhood/youth? |
| CJ28 | Was there any alcohol or drug abuse in the patients youth childhood/youth? |
| CJ29 | Did the patient fail at school in his childhood/youth? |
| PH1 | Age at which the F2x diagnosis was given? |
| PH2 | Age at which the patient showed first symptoms of the F2x diagnosis? |
| PH3 | delusions present in patients history? |
| PH4 | hallucinations present in patients history? |
| PH5 | penetrability of the own ego present in patients history? |
| PH6 | disorders of affect or drive present in patients history? |
| PH7 | negative symptoms present in patients history? |
| PH8 | Was the patient ever suicidal? |
| PH9 | Did the patient ever show any self-harming behavior? |
| PH10a | Did the patient ever attempt suicide? |
| PH11a | Did the patient ever show any endangerment of others? |
| PH12a | Was there ever any compulsory measure during a hospitalization used? |
| PH13 | Is there or was there any alcohol abuse? |
| PH14b | Cannabis abuse/-dependence? |
| PH14c | Opioid abuse/-dependence? |
| PH14d | Cocaine abuse/-dependence? |
| PH14e | consumption of stimulants, amphetamines, ecstasy? |
| PH15a | presence of a personality disorder (PD)? |
| PH16a | What was the personality structure of the patient (as described by pre-treating psychologist/psychiatrist)- paranoid? |
| PH16b | What was the personality structure of the patient (as described by pre-treating psychologist/psychiatrist)- schizoid? |
| PH16c | What was the personality structure of the patient (as described by pre-treating psychologist/psychiatrist)- dissocial? |
| PH16d | What was the personality structure of the patient (as described by pre-treating psychologist/psychiatrist)- emotional unstable? |
| PH16e | What was the personality structure of the patient (as described by pre-treating psychologist/psychiatrist)- anancastic? |
| PH16f | What was the personality structure of the patient (as described by pre-treating psychologist/psychiatrist)- dependent? |
| PH16g | What was the personality structure of the patient (as described by pre-treating psychologist/psychiatrist) - histrionic? |
| PH16h | What was the personality structure of the patient (as described by pre-treating psychologist/psychiatrist) - narcistic? |
| PH17a | Any other psychiatric/somatic comorbidity? |
| PH18a | Any outpatient psychiatric treatment(s) before investigated offence? |
| PH19a | Any inpatient psychiatric treatment(s) before investigated offence? |
| PH19c | If 1, number of inpatient treatment(s) before investigated offence? |
| PH22a | Was the patient ever under „FU“ or „FFE“ in inpatient psychiatric treatment before the investigated offence? |
| PH23a | Did the patient receive any neuroleptic medication before the investigated offence? |
| PH23u | If 1, was the neuroleptic medication consumed regularly before the investigated offence? |
| PH24a | Did the Patient receive no other medication? |
| PH24b | Did the Patient receive benzodiazepine? |
| PH24c | Did the Patient receive antidepressants? |
| PH24d | Did the Patient receive methylphenidate? |
| PH24e | Did the Patient receive mood stabilizer? |
| PH25a | Where there any problems in the treatment of the patient before the investigated offence / hospitalisation ? |
| PH25b | if 1 applies, medicative Noncompliance |
| PH25c | if 1 applies, Patient did not go to his ambulant psychiatrist |
| PH25d | if 1 applies, Patient consumed alcohol/drugs |
| PH25e | if 1 applies, Patient is homeless |
| PH25f | if 1 applies, Patient is not health insured |
| PH25g | if 1 applies, Patient was treated for another diagnosis |
| PH25h | if 1 applies, Patient was not in treatment |
| PH25i | if 1 applies, Patient refused therapy |
| CH1 | Are there any entries in the federal central criminal registry?(offence leading to forensic hospitalization excluded) |
| CH10a | Did a youth imprisonment occur? |
| N2 | Does/did the patient show an cognitive deficit? |
| N3 | Does/did the patient show any deficits in drive? |
| S1 | Does/did the patient have satisfying friendships? |
| S2 | Does/did the patient have a satisfying relationship to his parents? |
| S4 | Does/did the patient suffer under low self-esteem? |
| S5 | Does/did the patient suffer from social isolation? |
| S6 | Does/did the patient avoid women? |
| S8a | In which living situation did the patient spend most of his life- psychiatric accommodation measures? |
| S8b | In which living situation did the patient spend most of his life - complementary facility (dormitory, sheltered housing (with others ) |
| S8g | In which living situation did the patient spend most of his life - homeless? |
| S9a | Stressors in the recent past: relationship |
| S9b | Stressors in the recent past: as parents |
| S9c | Stressors in the recent past: job |
| S9d | Stressors in the recent past: financial issues |
| S9e | Stressors in the recent past: legal issues |
| S9f | Stressors in the recent past: own development |
| S9g | Stressors in the recent past: accident |
| S9h | Stressors in the recent past: illness |
| S9i | Stressors in the recent past: isolation |
| S9j | Stressors in the recent past: migration |
| S10 | Was the patient himself victim of a crime ? |
| DZ5 | Does/did the patient show no remorse or responsibility? |
| DZ7 | Does/did the patient show dis/antisocial utterances or attitudes? |
| DZ8 | Does/did the patient show no or minor willingness for therapy? |
| DZ10 | Does/did the patient constantly break rules? |
| DZ11 | Does/did the patient commit any substance abuse during the measure? |
| DZ12 | Does/did the patient show any failures during opening? |
| DZ13 | Does/did the patient show any unrealistic plans for his discharge? |
| R1a | Current psychiatric F2X diagnosis - schizophrenia? |
| R1b | Current psychiatric F2X diagnosis - hebephrenic schizophrenia? |
| R1c | Current psychiatric F2X diagnosis - acute psychotic disorder? |
| R1d | Current psychiatric F2X diagnosis - schizoaffective disorder? |
| R3 | Was the patient first diagnosed at admission? |
| R9e | Olanzapine equivalent at discharge |
| R9f | Polypharmacy at the time of discharge? |
| R10 | Was the patient ever suicidal during the current forensic hospitalization? |
| R11 | Did the patient ever show any self-harming behaviour during the current forensic hospitalization? |
| R12 | Did the patient ever attempt suicide during the current forensic hospitalization? |
| R13a | Did the patient ever show any endangerment of others during the current forensic hospitalization? |
| R14a | Was there ever any compulsory measure used during the current forensic hospitalization? |
| R27a | How can the results of the treatment concerning the psychiatric illness be evaluated? |
| PA1 | PANSS at admission: Scale Delusion |
| PA2 | PANSS at admission: Scale Conceptual disorganization |
| PA3 | PANSS at admission: Scale Hallucinations |
| PA4 | PANSS at admission: Scale Hyperactivity |
| PA5 | PANSS at admission: Scale Grandiosity |
| PA6 | PANSS at admission: Scale Suspiciousness?/persecution |
| PA7 | PANSS at admission: Scale Hostility |
| PA8 | PANSS at admission: Scale Blunted affect |
| PA9 | PANSS at admission: Scale Emotional withdrawal |
| PA10 | PANSS at admission: Scale Poor rapport |
| PA11 | PANSS at admission: Scale Passive?/apathetic social withdrawal |
| PA12 | PANSS at admission: Scale Difficulty in abstract thinking |
| PA13 | PANSS at admission: Scale Lack of spontaneity and flow of conversation |
| PA14 | PANSS at admission: Scale Stereotyped thinking |
| PA15 | PANSS at admission: Scale Somatic concern |
| PA16 | PANSS at admission: Scale Anxiety |
| PA17 | PANSS at admission: Scale Guilt feelings |
| PA18 | PANSS at admission: Scale Tension |
| PA19 | PANSS at admission: Scale Mannerisms and posturing |
| PA20 | PANSS at admission: Scale Depression |
| PA21 | PANSS at admission: Scale Motor retardation |
| PA22 | PANSS at admission: Scale Uncooperativeness |
| PA23 | PANSS at admission: Scale Unusual thought content |
| PA24 | PANSS at admission: Scale Disorientation |
| PA25 | PANSS at admission: Scale Poor attention |
| PA26 | PANSS at admission: Scale Lack of judgement and insight |
| PA27 | PANSS at admission: Scale Disturbance of volition |
| PA28 | PANSS at admission: Scale Poor impulse control |
| PA29 | PANSS at admission: Scale Preoccupation |
| PA30 | PANSS at admission: Scale Active social avoidance |
| PAA | PANNS Score at admission |
| PA32 | PANSS at discharge?/today: Scale Delusion |
| PA33 | PANSS at discharge?/today: Scale Conceptual disorganization |
| PA34 | PANSS at discharge?/today: Scale Hallucinations |
| PA35 | PANSS at discharge?/today: Scale Hyperactivity |
| PA36 | PANSS at discharge?/today: Scale Grandiosity |
| PA37 | PANSS at discharge?/today: Scale Suspiciousness?/persecution |
| PA38 | PANSS at discharge?/today: Scale Hostility |
| PA39 | PANSS at discharge?/today: Scale Blunted affect |
| PA40 | PANSS at discharge?/today: Scale Emotional withdrawal |
| PA41 | PANSS at discharge?/today: Scale Poor rapport |
| PA42 | PANSS at discharge?/today: Passive?/apathetic social withdrawal |
| PA43 | PANSS at discharge?/today: Scale Difficulty in abstract thinking |
| PA44 | PANSS at discharge?/today: Scale Lack of spontaneity and flow of conversation |
| PA45 | PANSS at discharge?/today: Scale Stereotyped thinking |
| PA46 | PANSS at discharge?/today: Scale Somatic concern |
| PA47 | PANSS at discharge?/today: Scale Anxiety |
| PA48 | PANSS at discharge?/today: Scale Guilt feelings |
| PA49 | PANSS at discharge?/today: Scale Tension |
| PA50 | PANSS at discharge?/today: Scale Mannerisms and posturing |
| PA51 | PANSS at discharge?/today: Scale Depression |
| PA52 | PANSS at discharge?/today: Scale Motor retardation |
| PA53 | PANSS at discharge?/today: Scale Uncooperativeness |
| PA54 | PANSS at discharge?/today: Scale Unusual thought content |
| PA55 | PANSS at discharge?/today: Scale Disorientation |
| PA56 | PANSS at discharge?/today: Scale Poor attention |
| PA57 | PANSS at discharge?/today: Scale Lack of judgement and insight |
| PA58 | PANSS at discharge?/today: Scale Disturbance of volition |
| PA59 | PANSS at discharge?/today: Scale Poor impulse control |
| PA60 | PANSS at discharge?/today: Scale Preoccupation |
| PA61 | PANSS at discharge?/today: Scale Active social avoidance |
| PAD | PANNS Score at discharge |
| PH1zuPH2 | Difference between age at which the patient showed first symptoms of the F2x diagnosis and age at which the F2x diagnosis was given |
| PAAzuPAD | Difference between PANNS Score at admission and PANNS Score at discharge |
| SD1zuPH2 | Difference between age at admission and age at which the patient showed first symptoms of the F2x diagnosis |

**References**

1. Kirchebner J, Machetanz L, Gunther M, Huber D, Lau S. Coding protocol Pathways into delinquency in offenders suffering from schizophrenia spectrum disorders [Internet]. 2022. Available from: https://www.researchgate.net/publication/363044110_Coding_protocol_Pathways_into_delinquency_in_offenders_suffering_from_schizophrenia_spectrum_disorders

2. Tanskanen J, Anttila T. A Prospective Study of Social Isolation, Loneliness, and Mortality in Finland. Am J Public Health. 2016 Nov;106(11):2042–8.
